# Effect of MDR1 gene polymorphisms on mortality in paraquat intoxicated patients

**DOI:** 10.1038/srep31765

**Published:** 2016-08-22

**Authors:** Hak Jae Kim, Hyung-Ki Kim, Jun-Tack Kwon, Sun-hyo Lee, Sam el Park, Hyo-Wook Gil, Ho-yeon Song, Sae-yong Hong

**Affiliations:** 1 Department of Clinical Pharmacology, College of Medicine, Soonchunhyang University, Cheonan, Choongcheongnam-do 330-930, Republic of Korea; 2 Department of Internal Medicine, Soonchunhyang University Cheonan Hospital, Cheonan, Republic of Korea; 3 Department of Microbiology, College of Medicine, Soonchunhyang University, Cheonan, Republic of Korea

## Abstract

Paraquat is a fatal herbicide following acute exposure. Previous studies have suggested that multidrug resistance protein 1 (MDR1) might help remove paraquat from the lungs and the kidney. MDR1 single-nucleotide polymorphisms (SNPs) are involved in the pharmacokinetics of many drugs. The purpose of this study was to determine whether MDR1 SNPs were associated with the mortality in paraquat intoxicated patients. We recruited 109 patients admitted with acute paraquat poisoning. They were genotyped for C1236T, G2677T/A, and C3435T single-nucleotide polymorphisms (SNPs) of MDR1 gene. Their effects on mortality of paraquat intoxicated patients were evaluated. Overall mortality rate was 66.1%. Regarding the C1236T of the MDR1 gene polymorphism, 21 (19.3%) had the wild type MDR1 while 88 (80.7%) had homozygous mutation. Regarding the C3435T MDR1 gene polymorphism, 37(33.9%) patients had the wild type, 23 (21.1%) had heterozygous mutation, and 49 (45.0%) had homozygous mutation. Regarding the G2677T/A MDR1 gene polymorphism, 38 (34.9%) patients had the wild type, 57 (52.3%) had heterozygous mutation, and 14 (12.8%) had homozygous mutation. None of the individual mutations or combination of mutations (two or three) of MDR1 SNP genotypes altered the morality rate. The mortality rate was not significantly different among SNP groups of patients with <4.0 μg/mL paraquat. In conclusion, MDR1 SNPs have no effect on the mortality rate of paraquat intoxicated patients.

Paraquat dichloride (1,1′-dimethyl-4,4′-bipyridinium dichloride, PQ) is a quick acting non-selective herbicide used to control broad leaf weeds. PQ intoxication is a serious public health problem. It has an estimated annual incidence of 2,000 toxic ingestions with mortality rates of 60–70% in some Asian countries^1^,^2^. Plasma PQ concentration reflects the degree of exposure. Although other prognostic markers have been studied, plasma PQ concentration has been considered as an important prognostic marker to predict mortality of PQ intoxicated patients^1^,^3^,^4^,^5^. Reducing PQ concentration might be important for patients.

P-glycoprotein (P-gp) is an efflux protein expressed in tumor tissues and a variety of normal tissues, including the blood-brain barrier, intestines, liver, and kidneys^6^,^7^,^8^. The inhibition or induction of P-gp can influence the pharmacokinetics of substrate drugs. Dexamethasone-induced P-gp expression decreases PQ accumulation in lung by increasing urinary and fecal excretion of PQ in Wister rats^9^. Methylprednisolone induces transmembrane ATP-dependent transporter P-gp expression that can greatly improve PQ-treated A549 cell viability, reduce accumulation of intracellular PQ, and prevent PQ induced cytotoxicity^10^. P-gp is located in the brush-border membrane of the kidney renal proximal tubules. It has been proposed that P-gp plays an important role in extruding xenobiotics and metabolic waste from the blood into the urine^11^. One study has shown that P-gp is involved in removing PQ from the kidneys and attenuating toxicity enhanced in MDR 1a/1b knockout mice^12^. Some reports have suggested that inducing P-gp might be used as a therapeutic approach to treat PQ intoxication^13^,^14^,^15^,^16^.

In recent years, >700 variations in nucleotide sequences of P-gp-encoding multidrug resistance 1 (MDR1) gene have been identified^17^. Some of them are associated with alterations in protein functions, consequently changing the pharmacokinetics of substrate drugs. The most extensively investigated MDR1 single-nucleotide polymorphisms (SNPs) are C1236T in exon 12, G2677T/A in exon 21, and C3435T in exon 26^18^. The frequencies of MDR1 SNPs are different among various ethics groups. The highest frequency of the homozygous mutant genotype (TT-TT-TT) in exon 12, 21, and 26 at positions 1236, 2677, and 3435 has been found in Indians (31%) followed by 19% in Chinese and 15% in Malays^19^. In Koreans, the frequencies of these MDR1 SNPs were 47.7% for C3435T, 37.6% for G2677T, 4.4% for G2677A, and 21.7% for T1236C^20^. The SNPs of MDR1 gene could influence pharmacokinetics, although the drugs themselves might not be associated with the expression of P-gp. Up to date, the relationship between SNPs of MDR1 gene and PQ kinetics remains unclear, although some studies have suggested that the function of P-gp could be associated with PQ intoxication. The relationship between MDR1 genotypes and mortality in PQ intoxicated patients have not been studied. Therefore, the objective of this study was to determine the effect of MDR1 SNPs on the mortality of PQ intoxicated patients.

## Methods

### Patients

We conducted a prospective study on PQ patients admitted from May 1, 2011 to December 30, 2011. This study included patients with positive urine sodium dithionite test and oral ingestion of PQ. The present study was approved by Soonchunghyang Cheonan Hospital’s Institutional Review Board. Informed consent was obtained from all subjects. Patients received standardized medical management according to PQ intoxication treatment guidelines used by Soonchunghyang University Cheonan Hospital. All treatments were performed by our trained physicians. Patients who were confirmed to have ingested PQ ≤ 2 h received gastric lavage, whereas those treated less than 12 h were administered with 100 g Fuller’s earth in 200 ml of 20% mannitol. Cyclophosphamide (15 mg/Kg) and methylprednisolone (1000 mg) were infused for 3 consecutive days. Antioxidant N-acetylcysteine (4 g) was infused for 7 days. Hemoperfusion was performed when result of urinary PQ test was positive.

### Data collection

Demographic variables such as age and sex of patients were recorded on a standardized data collection form. The time difference between the patient’s PQ exposure and arrival at Soonchunhyang University Cheonan Hospital was obtained from patient history. Clinical laboratory parameters (white blood cell count, hemoglobin, platelet, amylase, lipase, albumin, aspartate aminotransferase, alanine aminotransferase, total bilirubin, blood urea nitrogen, creatinine, pH, arterial oxygen concentration, arterial carbon dioxide concentration, and artrial bicarbonate) were obtained when patients arrived at the emergency unit.

Dithionite urine test were performed to quantitatively determine urine PQ levels. Ten milliliters of urine was placed into a beaker followed by the addition of 2 g of sodium bicarbonate. The mixture was shaken gently. One gram of sodium dithionite was then added to the mixture and the effervescence was allowed to subside. The mixture was then shaken again. Solid material was allowed to settle and the mixture was viewed against a white background. Results were presented in four grades: black (+4), deep blue (+3), light blue (+2), and barely distinguishable blue (+1). Based on our preliminary study, the cut-off value for PQ detection at grade (+1) was 1 μg/mL.

### MDR1 SNP Genotyping

Three MDR1 SNPs (rs2032582, rs1045642, and rs1128503) were genotyped in PQ intoxicated patients to evaluate the association between ATP-binding cassette sub-family B member 1 gene (*ABCB1*/MDR1) and PQ intoxication. These SNPs were selected from a previous study^21^ and the National Center for Biotechnology Information (NCBI) website (http://www.ensembl.org; www.ncbi.nlm.nih.gov/SNP). DNA was extracted from peripheral blood using a PureHelix Genomic DNA Prep kit (NanoHelix Co., Ltd., Daejeon, Korea) as described previously^22^.

Polymerase chain reaction-restriction fragment length polymorphism (PCR-RFLP) protocols are summarized in Table 1. Primer sequences and annealing temperatures used for the analysis of each polymorphism are also listed in Table 1. Each reaction consisted of a single denaturation step at 95 °C for 5 min, 35 cycles of denaturation at 95 °C for 30 sec, annealing with appropriate primer pair at annealing temperature for 30 sec, and extension at 72 °C for 30 sec. A final extension step at 72 °C was performed at the end of the PCR program for 10 min. Following PCR amplification, products were digested overnight with corresponding restriction enzymes (Table 1) according to the manufacturer’s instructions. The digested products were electrophoresed on 3.0% agarose gels and stained with SYBR-Green (Invitrogen, Carlsbad, CA, USA). All restriction enzymes used in this study were purchased from New England Biolabs (Ipswich, MA, USA). Reproducibility of genotyping was assured by conducting duplicate experiments. Genotype analysis was conducted by blinding the case and control status.

### Statistical analysis

Continuous variables are expressed as mean ± standard deviation with or without the median value and range. Categorical variables are shown as frequencies (number of cases and percentages). Differences between groups were detected using chi-square test or Fisher’s exact test for categorical variables. Binary logistic regression analysis was used to identify the risk of mortality according to gene polymorphism. Results of the logistic regression analyses are reported as relative risks or odds ratios with 95% confidence intervals. Statistical analyses were performed using SPSS ver. 14.0 software (SPSS Inc., Chicago, IL, USA). Statistical significance was considered when *P*-value was less than 0.05.

## Results

### Baseline characteristics of the study population

A total of 109 patients were included in this study. Baseline laboratory parameters and initial patient information recorded at hospital arrival are summarized in Table 2. Overall mortality rate was 66.1%, similar to the mortalities rates in previous studies^1^,^23^. Semi-quantitative dithionite tests revealed that 27 patients had grade 1+ (blue color), 22 patients had grade 2+ (tender blue), and 60 patients had grade 3+ (dark blue) paraquat levels. The mean time to death was 4.67 ± 5.11 days. The frequencies of the three MDR1 SNP genotypes (C3435T, C1236T, and G2677T/A) in PQ intoxicated patients are shown in Table 3.

### Effect of MDR1 gene polymorphisms on mortality

Regarding the frequencies of C1236T MDR1 gene polymorphism, 21 (19.3%) patients had the wild type, 88 (80.7%) patients had the homozygous mutation, and none had the heterozygous mutation. The frequencies of C1236T MDR1 polymorphism in survivors were not significantly different from those in non-survivors (χ^2^ = 3.604, *P*-value = 0.165). Regarding the frequencies of C3435T MDR1 polymorphism, 37 (33.9%) patients had the wild type, 23 (21.1%) patients had heterozygous mutation, and 49 (45.0%) patients had homozygous mutation. The frequency distributions of the C3435T MDR1 SNP were not significantly different between survivors and non-survivors either (χ^2^ = 0.004, *P*-value = 0.947). Regarding the frequencies of G2677T/A MDR-1 polymorphism, 38 (34.9%) patients had the wild type, 57 (52.3%) patients had the heterozygous mutation, and 14 (12.8%) patients had homozygous mutation. The frequency distributions of the C2677T/A MDR1 polymorphism were not significantly different between survivors and non-survivors (χ^2^ = 1.506, *P*-value = 0.471). Neither heterozygous nor homozygous mutation of the three MDR1 gene polymorphisms had any effect on the mortality of PQ intoxicated patients (Table 3).

### Effect of MDR1 gene polymorphism on mortality in patients with <4.0 μg/mL paraquat

Patients with high plasma PQ concentrations were included in this study. We reanalyzed the 67 patients with <4.0 μg/mL PQ concentration. However, none of the mutations in MDR1 gene affected the mortality of these patients (Table 4). The genotype frequencies of MDR1 SNPs were not associated with mortality either.

### Synergistic effect of two or three MDR1 SNP genotypes on mortality

We analyzed the frequency distributions of genotypes of two or three MDR1 SNPs between survivors and non-survivors to investigate the synergistic effect of two or three MDR1 SNPs on mortality (Table 5). When the effect of two or three SNPs in different combinations was evaluated among patients with wild or heterozygous mutant (represented as 0) or homozygous mutant genotype (represented as 1), the frequencies of the SNP combinations bearing the mutant genotype were not significantly different between survivors and non-survivors.

## Discussion

P-gp is a glycosylated membrane-bound efflux pump protein that removes substrates from the inside to the outside of the cell. Some reports have shown that inducing P-gp can protect cells against PQ induced toxicity *in vivo* and *in vitro*^9^,^10^,^12^,^13^,^14^,^15^. Silva *et al.* have demonstrated that inducing P-gp in Caco-2 cells using newly synthesized thioxanthones can prevent PQ cytotoxicity^15^. Mice treated with dexamethasone display increased MDR1 expression in the lungs associated with decreased PQ accumulation and pneumotoxicity^9^. In addition to the lungs and liver, MDR1 and Mdr1a/1b are also expressed in human and rodent kidneys, respectively^24^,^25^. Xia *et al.* have shown MDR1/Mdr1 participates in the elimination of PQ from the kidneys and protects against subsequent toxicity^12^. These results suggest that P-gp is involved in the PQ intoxication mechanisms.

MDR1 gene polymorphisms have been associated with altered drug absorption, disposition, and toxicity responses^26^. Among MDR1SNPs, C1236T in exon 12, G2677T/A in exon 21, and C3435T in exon 26 have been investigated extensively. For example, renal transplant recipients with homozygous mutation in G2677T/A require higher tacrolimus dose than recipients without such mutation to receive the same therapeutic effect^27^. The MDR1 C3435T and G2677T/A polymorphisms are risk factors for increased susceptibility to nephrotic syndrome and steroid resistance^28^. It is currently unclear whether MDR1 genetic polymorphisms can affect the pharmacokinetics and toxicities of PQ. However, variations in MDR1 expression between individuals may alter susceptibility to PQ-induced toxicity. This is the first study to investigate the effect of MDR1 SNPs on mortality of PQ intoxicated patients. In this study, the frequency distributions of homozygous and heterozygous mutation for the G2677T/A, C3435T, and C1236T SNPs were not different between non-survivors and survivors of PQ toxicity. We reanalyzed these patients with a PQ concentration <4 μg/mL because our previous report showed that patients with >4 μg/mL could not survive^29^. However, the frequencies of MDR1 genotypes did not affect the mortality rates of these patients. In addition, two or three SNPs in different combinations did not exhibit significant difference in the mortality of these patients.

However, this study has some limitations. First, our treatment protocol included in the dexamethasone infusion might have affected the function of lung P-gp regardless of the MDR1 polymorphism. Second, we could not show pharmacokinetics changes according to MDR1 gene SNPs. Third, our study population included more mutants compared to that of a previous study. The C1236T frequencies in Asians are 8.3–13.8% of wild type (CC), 37.9–44.6% (CT) of heterozygous, and 43.5–52.1% of homozygous mutants (TT)^30^,^31^. The CT type was not included in our study, which might have produced selection bias. Fourth, P-gp expression in human organ might be different compared to that in rodents. Therefore, these gene polymorphisms of MDR1 might be able to influence the P-gp function of kidney and lung. Although P-gp in human lung is expressed in alveolar epithelia cell type 1^32^, it is necessary to reveal the relationship between SNPs and P-gp function in the lung in the future.

In conclusion, our observations suggest that the MDR1 SNPs do not have any effect on the mortality of PQ intoxicated patients.

## Additional Information

**How to cite this article**: Kim, H. J. *et al.* Effect of MDR1 gene polymorphisms on mortality in paraquat intoxicated patients. *Sci. Rep.* **6**, 31765; doi: 10.1038/srep31765 (2016).

## Figures and Tables

**Table 1:** Genotyping conditions for the MDR1 gene polymorphisms.

SNP	Primer sequence	Annealing temperature (°C)	Enzyme	Cleavage products(bp)
C1236T (rs1128503)	F:TATCCTGTGTCTGTGAATTGCC	54	HaeIII	370 (272, 98/272, 98, 63, 35/272, 63, 35)
	R:CCTGACTCACCACACCAATG			
C3435T (rs1045642)	F:TGTTTTCAGCTGCTTGATGG	53	San3AI	197 (197/197, 158, 39/158, 39)
	R:AAGGCATGTATGTTGGCCTC			
G2677T (rs2032582T)	F:TGCAGGCTATAGGTTCCAGG	58	BanI	224 (224/224, 198, 26/198, 26)
	R:TTTAGTTTGACTCACCTTCCCG			
G2677A (rs2032582A)	F:TGCAGGCTATAGGTTCCAGG	58	BsrI	220 (220/220, 206, 14/206, 14)
	R:GTTTGACTCACCTTCCCAG			

**Table 2:** Clinical characteristics of the 109 paraquat intoxicated patients.

		Reference value
Age (years)	54.6 ± 16.4	
Men (cases)	68 (62.4%)	
Estimated amount of paraquat dichloride (24.5% concentration) exposure (mL)	86.5 [5, 300]*	
Time interval between exposure and hospital admission (hours)	11.7 ± 14.7	
Plasma paraquat level (μg/mL)	29.24[0.01, 618.66]*	
Mortality (cases)	72 (66.1%)	
White blood cell (/μl)	15552.0 ± 7633.6	4000–10800
Hemoglobin (g/dl)	13.7 ± 2.1	12–16
Platelet (*10^3^/μl)	248.8 ± 100.4	130–400
Albumin (g/dl)	4.3 ± 0.7	3.1–5.2
Total bilirubin (mg/dl)	0.8 ± 0.7	0.2–1.2
Aspartate aminotransferase (IU/L)	49.8 ± 96.7	0–40
Alanine transaminase (IU/L)	30.6 ± 41.1	0–40
Amylase (IU/L)	268.9 ± 442.3	28–110
Lipase (IU/L)	100.2 ± 280.0	0–60
Blood urea nitrogen (mg/dl)	18.0 ± 13.0	8–20
Creatinine (mg/dl)	1.5 ± 1.4	0.5–1.2
Sodium (mEq/L)	140.4 ± 7.6	136–145
Chloride (mEq/L)	101.5 ± 7.3	96–110
Potassium (mEq/L)	3.6 ± 0.8	3.5–5.1
Uric acid (mg/dL)	5.2 ± 1.9	3.0–7.0
pH	7.4 ± 0.1	7.35–7.45
pCO_2_ (mmHg)	30.5 ± 7.5	32–45
pO_2_ (mmHg)	91.4 ± 19.2	75–100
HCO_3_^−^ (mEq/L)	18.5 ± 5.9	

Values are expressed as mean ± standard deviation (percentage) or *median [minimum, maximum].

**Table 3:** Distribution of the MDR1 gene polymorphisms in paraquat intoxicated patients.

SNP	Survival N(%)	Non-survival N(%)	OR	95% CI	P-value
C1236T					
CC	7 (18.9%)	14 (19.4%)			
TT	30 (81.1%)	58 (80.6%)	0.967	0.353–2.655	0.947
C3435T					
CC	15 (40.5%)	22 (30.6%)			
CT	10 (27%)	13 (18.1%)	0.866	0.309–2.542	0.822
TT	12 (32.4%)	37 (51.4%)	2.102	0.834–5.299	0.155
CT + TT	22 (59.4%)	50 (69.5%)	1.155	0.678–3.539	0.299
G2677T/A					
GG	15 (40.5%)	23 (31.9%)			
GT or A	19 (51.4%)	38 (52.8%)	1.304	0.556–3.059	0.541
TT or A	3 (8.1%)	11 (15.3%)	2.391	0.571–10.020	0.233
GT or A + TT or A	22 (59.5%)	49 (68.1%)	1.453	0.638–3.306	0.374

**Table 4:** Distribution of the MDR-1 gene polymorphisms in patients with <4.0 μg/mL plasma paraquat concentration.

SNP	Survival N(%)	non-survival N(%)	OR	95% CI	P-value
C1236T					
CC	7 (19.4%)	7 (22.6%)			
TT	29 (80.6%)	24 (77.4%)	0.828	0.255–2.691	0.753
C3435T					
CC	14 (38.9%)	10 (32.3%)			
CT	10 (27.8%)	7 (22.6%)	0.98	0.278–3.460	0.975
TT	12 (33.3%)	14 (45.2%)	1.633	0.533–5.003	0.39
CT + TT	22 (61.1%)	21(67.8%)	1.336	0.488–3.662	0.573
G2677T/A					
GG	15 (41.7%)	13 (41.9%)			
GT or A	18 (50.0%)	11 (35.5%)	0.705	0.245–2.026	0.517
TT or A	3 (8.3%)	7 (22.6%)	2.692	0.575–12.596	0.208
GT or A + TT or A	21 (58.3%)	18 (58.1%)	0.989	0.374–2.618	0.982

**Table 5:** Combined distribution of the MDR-1 gene polymorphisms in paraquat intoxicated patients.

	Survival N(%)	non-survival N(%)	OR	95% CI	P-value
C3435T and C1236T					
C3435T(0) + C1236T(0)	6	11			
C3435T(1) + C1235T(0)	1	3	1.636	0.138–19.387	0.696
C3435T(0) + C1235T(1)	30	58	1.055	0.355–3.130	0.924
C3435T and G2677TA					
C3435T(0) + G2677TA(0)	24	32			
C3435T(1) + G2677TA(0)	10	29	2.175	0.891–5.310	0.088
C3435T(0) + G2677TA(1)	3	11	2.750	0.690–10.952	0.151
C1236T and G2677TA					
C1236T(0) + G2677TA(0)	7	12			
C1236T(1) + G2677TA(0)	27	49	1.059	0.373–3.007	0.915
C1236T(0) + G2677TA(1)	3	11	2.139	0.440–10.391	0.346
C3677T, C1236T and G2677TA					
C3677T(0) + C1236T(0) + G2677TA(0)	6	9			
C3677T(1) + C1236T(0) + G2677TA(0)	1	3	2.000	0.166–24.069	0.585
C3677T(0) + C1236T(0) + G2677TA(1)	0	2			
C3677T(0) + C1236T(1) + G2677TA(0)	18	23	0.852	0.256–2.837	0.794
C3677T(1) + C1236T(0) + G2677TA(1)	1	1	0.667	0.035–12.840	0.788
C3677T(1) + C1236T(1) + G2677TA(0)	9	26	1.926	0.535–6.936	0.316
C3677T(1) + C1236T(1) + G2677TA(1)	2	8	2.667	0.414–17.169	0.302
